# A Comparative Pharmacokinetic Analysis of Oral and Subcutaneous Meloxicam Administered to Postpartum Dairy Cows

**DOI:** 10.3390/vetsci6030073

**Published:** 2019-09-05

**Authors:** Daniel Shock, Steven Roche, Merle Olson

**Affiliations:** 1 ACER Consulting, Guelph, ON N1G 5L3, Canada; 2 Alberta Veterinary Laboratories, Calgary, AB T2E 6J7, Canada

**Keywords:** dairy cow, meloxicam, pharmacokinetics, non-steroidal anti-inflammatory, early lactation

## Abstract

The dairy industry needs evidence-based solutions to mitigate painful procedures and conditions in dairy cattle. The objective of this study was to compare the pharmacokinetic properties of orally versus subcutaneously administered meloxicam in early-lactation dairy cattle. The study was conducted at a commercial dairy herd in southwestern Ontario, Canada. Twelve postpartum cows were enrolled in the study, receiving either subcutaneous meloxicam (MET) at 0.5 mg/kg body weight (n = 6) or oral meloxicam (MOS) at a higher dose of 1.0 mg/kg body weight (n = 6) immediately following parturition. The predicted half-life (12.5 ± 2.0 vs. 28.5 ± 2.0 h), C_max_ (1.59 ± 0.15 vs. 1.95 ± 0.16 μg/mL), T_max_ (5.33 vs. 11.7 h), and AUC_0→∞_ (39.6 ± 7.4 vs. 115.6 ± 19 h * µg/mL) differed significantly between MET and MOS cows, respectively. After controlling for the treatment group, first lactation cows had a significantly higher half-life (4.1 ± 2.1 h), C_max_ (0.56 ± 0.2 µg/mL), and AUC_0→∞_ (21.6 ± h * µg/mL) relative to second lactation or greater cows, respectively. Administration of meloxicam through the subcutaneous or oral route results in appreciable, dose-dependent systemic levels.

## 1. Introduction

As the dairy industry looks to ensure its long-term sustainability, animal welfare considerations need to be addressed to maintain and improve consumer support [1]. As an understanding of how pain is experienced in animals evolves, there are several common conditions and procedures in the dairy industry where pain has historically not been adequately controlled [2]. The dairy industry needs evidence-based solutions to mitigate painful procedures and conditions.

Meloxicam is a selective cox-2 non-steroidal anti-inflammatory (NSAID) drug approved for the treatment of inflammatory and/or painful conditions in a variety of species [3]. In cattle, meloxicam has shown efficacy treating the pain and inflammation associated with many conditions, namely castration [4], dehorning [5,6], surgery [7], mastitis [8], dystocia [9,10], and diarrhea [11].

The pharmacokinetics of oral meloxicam in dairy cattle have been studied [12,13,14]; however, there have been no published reports evaluating the pharmacokinetics of a novel oral meloxicam product, meloxicam oral solution (MOS, 15 mg/mL, Alberta Veterinary Laboratories, Calgary, Canada). MOS is approved as a single oral administration in Canada for the alleviation of pain and inflammation following surgical and band castration in cattle. To date, there is no published literature evaluating the pharmacokinetic properties of meloxicam in this liquid form in mature cattle. Pharmacokinetics human meloxicam pills administered to lactating dairy cattle has been reported [15]. Further, although the pharmacokinetics of subcutaneously administered meloxicam have been studied in goats [16], the authors are not aware of published data in cattle. Metacam (MET, 20 mg meloxicam/mL solution, Boehringer Ingelheim Canada, Burlington, ON, Canada) is approved in Canada as a single subcutaneous injection for the symptomatic treatment of inflammation and pain associated with acute clinical mastitis, and for the reduction in pain associated with abdominal surgery in adult dairy cattle.

The objective of the current study is to evaluate the pharmacokinetic properties of orally and subcutaneously administered meloxicam in early-lactation dairy cattle. It is hypothesized that oral meloxicam will have a longer half-life, higher area under the curve, more muted maximal plasma concentration, and slower elimination rate relative to subcutaneously administered meloxicam.

## 2. Materials and Methods

### 2.1. Ethics Statement

The present field-based study was carried out in accordance with the recommendations of the Guide to Care and Use of Farm Animals in Research, Teaching, and Testing set forth by the Canadian Council on Animal Care. The protocol was reviewed and approved by the Alberta Agriculture Institutional Animal Care and Use Committee (Airdrie, AB, Canada, study number: AVL18007).

### 2.2. Study Herd and Treatment Protocol

The study was conducted at a commercial dairy farm milking 200 cows in southwestern Ontario, Canada, between October and November 2018. Average milk production per cow was 43 kg, at 4.2% fat and 3.4% protein, at an average number of days in milk of 147.

Cows were randomly allocated to receive the manufacturer label dose of either MOS (1 mg/kg orally) or MET (0.5 mg/kg subcutaneously). The farm staff were responsible for medication administration, blood sample collection and storage, and reporting any disease events. There was no formal sample size calculation performed prior to study commencement. Last, there was no attempt to stratify treatment allocation by lactation group to ensure even distribution of potential confounders.

Treatments were administered immediately following calving. Blood samples were collected via venipuncture of the coccygeal vein into heparinized tubes. Blood was taken at 0 (pre-medication), 2, 4, 8, 12, 24, 36, 48, and 72 h relative to treatment administration. Samples were centrifuged (within 1-h post-collection) for 20 min at 2000× *g* in a temperature-controlled (approximately 5 °C) centrifuge. Plasma samples were then aliquoted into prelabelled (Study Number, Sample number, animal ID #, date and study time) plastic vials. The vials were stored at −20 °C until submitted for analysis.

### 2.3. Laboratory Analysis for Meloxicam Quantitation in Plasma

Processed plasma samples received at the diagnostic laboratory (Alberta Veterinary Laboratories, Calgary, AL, Canada) were rapidly transferred to frozen storage (−20 °C). Storage temperature was monitored by a National Institute of Standards in Technology (NIST)-certified temperature-recording device.

Plasma samples were analyzed with a validated high-performance liquid chromatography (HPLC; Agilent 1200 HPLC, Agilent, CA, USA) procedure that was conducted using UV detection. An internal standard (piroxicam) was added to the untreated plasma sample. Standard concentrations used were 0.05, 0.1, 0.5, 1, 2, 3, 4, and 6 µg/mL respectively. Meloxicam and the internal standard were then extracted from plasma by solid-phase extraction (SPE). The SPE cartridges were connected to a vacuum manifold and conditioned with 1 mL methanol followed by 1 mL of water. The samples were passed through sorbents at a flow rate of less than 1 mL/min. The cartridges were then rinsed with 1 mL of 5% methanol and dried under vacuum for 2 min. Analytes were eluted with 1.5 mL of methanol. The eluent was then dried under vacuum at 40 °C for 2 h and the dried residue was reconstituted in 100 µL of mobile phase. The reconstituted sample was vortexed for 15 s then centrifuged at 14,000× *g* for 10 min to remove any particulate from the sample. Following centrifugation, 10 µL of supernatant was injected into the HPLC system. HPLC apparatus consisted of a pump system equipped with an automatic injector and UV detector (360 nm). Separation was achieved using a reverse-phase column (C18, 3 mm, 125 × 3.0 mm) and a guard column (C18, 10 × 30 mm). The mobile phase consisted of a mixture of acetic acid: 1% methanol (40:60) at a flow rate of 0.4 mL/min. For these conditions, meloxicam and piroxicam were eluted at a retention time of 7.8 and 5.4 min, respectively. Results for the method were linear over the calibration range of 10 to 1250 ng/mL as determined by use of a weighted linear regression model. Within-day and day-day precision were <10%. Accuracy ranged from 96% to 99%. The validated limit of quantification (LOQ) was 10 ng/mL.

### 2.4. Data Collection and Analysis

Variables of interest for analysis included date and time of calving, time of meloxicam administration and blood collection, lactation number, disease information, and milk production. Variables of interest were downloaded from the commercial herd management software (DairyComp 305, VAS, Tulare, CA, USA). All datasets were imported as comma-separated files (Table S1) into the Stata 14 statistical software program (StataCorp LP, College Station, TX, USA) for statistical analysis. The cow was considered the unit of analysis for this study.

Pharmacokinetic parameters were estimated for each animal using non-compartmental analysis using PKSolver, a validated Microsoft Excel add-on [17]. The maximum plasma concentration (C_max_) and time taken to reach maximum concentration (T_max_) were determined directly from the data. The area under the curve from 0 to last measurement (AUC_0→last_), AUC from 0 to infinity (AUC_0→∞,_ estimated with the linear fit of the natural log using the trapezoidal rule), the meloxicam plasma half-life (T_1/2_), and elimination rate (ke) were subsequently calculated for each cow. To estimate meloxicam concentration over time (i.e., between sampling intervals), the following Equation (1) was used:

(1) $$C_{i+1j}=\;C_{ij}-\left(C_{ij\;}*\;KE_{j}\right)$$

where *C_ij_* is the drug concentration for the *j*th cow at experimental hour *i*, *KE_j_* is the elimination rate constant for the ith cow, and *C_i+1j_* is the new meloxicam concentration for the *j*th cow at time (hour) *i* + 1. Note, the first concentration used for this equation (*C*_0_) is *C*_max_, where the elimination rate constant serves to estimate the plasma elimination of meloxicam over time. The resultant concentration approximations were used to estimate time above plasma concentrations associated with clinical analgesic and anti-inflammatory efficacy.

Descriptive statistics were generated for the final dataset for the pharmacokinetic outcomes of interest, including *C*_max_, T_max_, AUC_0→last_, AUC_0→∞_, T_1/2_, and KE. Further, descriptive analysis of cow demographics (parity and milk production) was summarized by the treatment group.

Univariable linear regression modelling was employed to study variables of interest. Statistical comparisons were made between potential predictor variables (including MOS vs. MET) and outcomes of interest. For this study, the outcomes of interest were C_max,_ T_max_, AUC_0→∞_, and T_1/2_. Variables explored for each model included: The treatment group (MOS vs. MET), lactation group (2 and over 3), and the non-lactating days prior to parturition group. Variables that had moderate statistical associations with the outcome of interest (defined at a liberal *p*-value < 0.2) were included in subsequent multivariable models.

For plasma meloxicam pharmacokinetics, a repeated measures linear mixed model [18] was constructed using cow over sampling time as a random effect and treatment (MOS vs. MET) and parity as fixed effects. Linear regression models were constructed for the pharmacokinetic outcomes. A stepwise backwards elimination process was employed, where variables identified as potentially associated with the outcome of interest in the initial univariable screening were included in a full multivariable model. Those variables having *p*-values for partial F-tests or type III tests of fixed effects greater than 0.05 were eliminated from the model after assessing whether they were part of biologically plausible interaction terms or had a confounding effect on the outcome of interest (e.g., a > 20% change in coefficient values when the term is removed from the model). Continuous variables were assessed for linearity with predicted model outcomes through visually inspecting a locally weighted scatterplot smoothing (LOWESS) curve for linear relationship, as well as the significance (*p* < 0.05) of a quadratic term in the model. If a continuous variable did not have a linear relationship with the outcome of interest, it was subsequently categorized based on biologically relevant cut-points, or if appropriate, a quadratic term was retained in the model. Variables retained in the final model were assessed for collinearity through the examination of Pearson or Spearman rank-order correlation coefficients. When high correlation was found between variables (>0.6), the most biologically appropriate variable was chosen for inclusion in the final model. Standardized residuals were generated and visually assessed for normality and homoscedasticity for the linear mixed regression model. If the residuals were heteroskedastic or not normally distributed, appropriate transformations were performed on the outcome of interest.

## 3. Results and Discussion

Table 1 outlines the lactation distribution and milk production level of study cows across treatment groups. The MOS group consisted of one more first lactation animal than the MET group. This difference in treatment allocation, driven by a lack of stratified randomization, likely contributed to the higher level of milk production noted in the MET group. There was no clinical disease in study animals during the duration of the study period.

**Figure 1:**
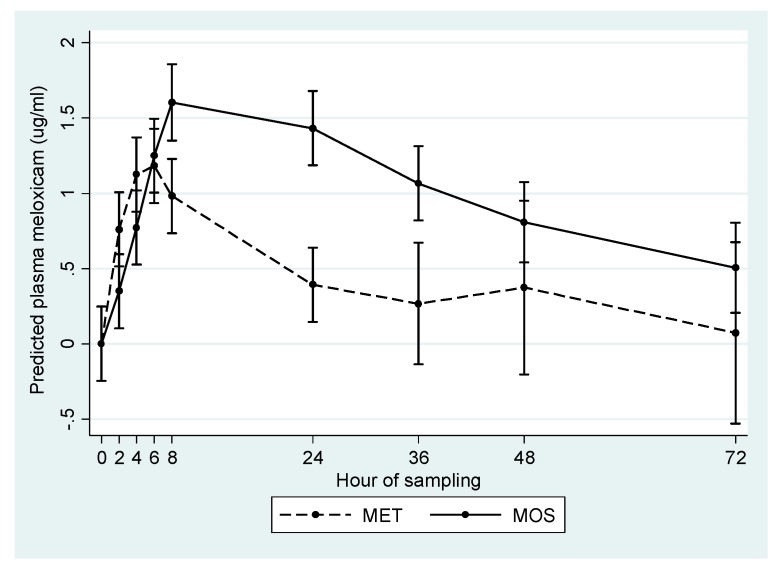
Predicted mean plasma meloxicam concentration (µg/mL) in cows treated orally (MOS) or subcutaneously (MET) after calving.

The predicted concentration-time profile of MET and MOS groups, after controlling for parity and repeated measures over time, is presented in Figure 1. Additionally, calculated pharmacokinetic parameters for study animals are presented in Table 2. Overall, plasma meloxicam levels were higher and lasted for a longer period in the MOS group relative to the MET group.

When assessing these relationships in multivariable linear modelling, parity played a significant role in the pharmacokinetic properties of plasma meloxicam. First lactation cows had significantly higher blood levels for longer periods of time relative to mature cows in both treatment groups (Table 3, Table 4 and Table 5). First lactation cows have significantly different metabolic and endocrine profiles relative to mature animals, likely driven by lower milk production and the fact that they are still growing [19]. This study highlights the need to consider parity when administering NSAID drugs. Specifically, due to concerns with renal damage and abomasal ulceration [3], caution is warranted when dosing primiparous animals due to their prolonged systemic drug levels.

Further, MOS-treated cows had significantly higher AUC_0→∞_, half-life, and C_max_ relative to MET cows (Table 3, Table 4 and Table 5). This is not surprising, given that the oral dose employed in the current study (1 mg/kg bodyweight) was double that of subcutaneous meloxicam (0.5 mg/kg bodyweight).

Few studies have evaluated plasma meloxicam levels associated with a clinical response, with none of them having been conducted in cattle. Based on previous research in equine and canine species, the EC_50_ (effective plasma concentration) of meloxicam varies between 0.2–0.7 µg/mL [20,21,22,23]. Using the low and high end of these plasma thresholds, the expected clinical therapeutic duration of MET and MOS are presented in Table 6. Briefly, MOS-treated cows had therapeutic plasma concentrations between 30–93 h; whereas, MET-treated cows had therapeutic plasma concentrations between 14–46 h using thresholds of 0.7 and 0.2 µg/mL. Caution should be used when interpreting the durations in Table 6, as these thresholds were chosen based on studies for conditions in other species. Future research should focus on establishing EC_50_ levels for painful conditions in cattle before definitive conclusions can be drawn.

To the author’s knowledge, this is the first study to evaluate the pharmacokinetic parameters of meloxicam given via subcutaneous route in mature dairy cattle. Previous research has studied subcutaneous administration of meloxicam in goats [16]. The predicted half-life (12.5 ± 2.02 vs. 15.16 ± 4.74 h), C_max_ (1.59 ± 0.15 vs. 1.91 ± 0.39 µg/mL), and T_max_ (5.33 vs. 3.20 ± 1.64 h), and AUC_0→∞_ (39.6 ± 7.4 vs. 24.65 ± 5.71 h * µg/mL) varied between cattle (current study—Table 3, Table 4 and Table 5) and goats [16], respectively. Goats metabolize drugs at a faster rate than cattle [24], therefore it is surprising to see the discrepancy between reported half-life parameters. Meloxicam did reach maximal plasma concentration 2 h earlier, with an overall lower AUC_0→∞_ noted in the goat study.

Other studies have evaluated the pharmacokinetics of parenteral (intravenous) meloxicam in immature ruminating cattle [12,20]. Both studies reported longer half-lives (19 and 21 h, respectively) and a greater AUC_0→∞_ (89 and 81 h * µg/mL, respectively) than found in the current study [12,20] (Table 2, Table 3, Table 4 and Table 5). In several studies young calves consistently have a longer half-life than older animals for both oral and injectable meloxicam [12,13,14,15,16]. It is difficult to make definitive conclusions, given that the current study assessed pharmacokinetic parameters in adult dairy cattle in very early lactation; whereas, the cited studies were conducted in 3- to 5-month old, weaned and growing Holstein calves. The sample size (n = 6) in this study was low, making definitive conclusions challenging. Further larger studies are warranted to understand whether differences in pharmacokinetic parameters between intravenous and subcutaneous administrations were real or due to random chance.

Oral administration of meloxicam tablets has been studied in ruminants. Relative to a previous study that administered meloxicam orally to ruminant calves at a dose of 0.5 mg/kg, MOS-treated cows had a longer half-life (28.5 ± 2.0 vs. 20.5 h) and T_max_ (13.3 vs. 11.7 h), a similar AUC_0→∞_ (115.6 ± 19 vs. 90.5 h * µg/mL), and markedly lower C_max_ (1.95 ± 0.16 vs. 2.9 µg/mL), respectively [25]. This study used a lower dose of meloxicam (0.5 mg/kg vs. the 1 mg/kg of the current study) in a younger class of cattle (non-lactating calves), with the meloxicam in tablet form. These factors could have led to differences in systemic absorbance and drug elimination.

Another study specifically evaluating the pharmacokinetic properties of oral meloxicam (1 mg/kg) in mature, postpartum cows noted higher C_max_ (2.92 µg/mL), T_max_ (17.60 h), with similar AUC_0→∞_ (109.34 h * µg/mL) parameters [15] relative to the current study (Table 2, Table 3, Table 4 and Table 5). Note that this study employed a tabular formulation of meloxicam, potentially contributing to differing absorption rates relative to the liquid meloxicam formulation evaluated in the current study.

The pharmacological preparations in this study were administered to healthy cows soon after parturition. While medication of healthy animals is difficult to justify, there is mounting evidence that parturition is painful in cattle [26,27]. The use of NSAID products in cows following calving has promoted greater intakes [28], alterations in activity level [10], higher milk production [29,30], lower somatic cell counts [26], and culling rates [30] in immediate post-partum period. Given the increased public scrutiny faced by the industry over issues of animal welfare [31], there may come a time when pain control is given to every cow following parturition.

## 4. Conclusions

To the authors knowledge, this is the first study to have evaluated the pharmacokinetic properties of subcutaneously administered meloxicam in early lactation, adult dairy cattle. Overall, both drugs (oral and subcutaneous meloxicam products) attained systemic therapeutic concentrations. This study also highlights the importance of evaluating pharmacokinetic parameters in both first lactation and mature lactating cows, as the pharmacokinetics can differ markedly. The oral meloxicam product had significantly longer half-life and T_max_, as well as higher AUC_0→∞_, C_max,_ and KE parameters relative to the parenteral product. These differences were likely driven by a more prolonged absorption, as well as a higher absolute dosage, associated with oral meloxicam treatment.

## Figures and Tables

**Table 1:** Descriptive statistics of study cows.

Variable	Oral Meloxicam (MOS)	Subcutaneous Meloxicam (MET)	Total
**Parity**			
**# 1st lactation cows**	3	2	5
**# ≥2nd lactation cows**	3	4	7
**Kg milk production (Std. Dev.) at first test**	39 (8.6)	43 (12.5)	41 (10.9)

**Table 2:** Pharmacokinetic parameters of plasma meloxicam in oral- and subcutaneously-treated cows.

	MOS ^1^		MET ^2^	
	Mean (SE ^9^)	Range	Mean (SE)	Range
**Half Life ^3^**	25.71 ^a^ (1.39)	18.57–32.68	8.95 ^b^ (1.85)	4.11–12.37
**AUC_0-t_^4^**	67.46 ^a^ (14.25)	25.52–113.26	22.26 ^b^ (4.70)	14.84–45.04
**AUC_0-∞_^5^**	86.13 ^a^ (17.46)	39.89–150.05	25.53 ^b^ (5.11)	15.31–46.65
**C_max_^6^**	1.68 ^a^ (0.20)	1.08–2.30	1.22 ^a^ (0.10)	0.76–1.47
**T_max_^7^**	13.33 ^a^ (3.37)	8–24	5.33 ^b^ (0.42)	4–6
**KE ^8^**	0.03 ^a^ (0.001)	0.02–0.04	0.09 ^b^ (0.02)	0.05–0.17

^1^ Meloxicam Oral Solution; ^2^ Metacam; ^3^ hours (h); ^4^ area under the curve from 0 to last time (h * µg/mL); ^5^ area under the curve from 0 to infinity; ^6^ maximum plasma concentration (µg/mL); ^7^ time at maximum plasma concentration (h); ^8^ elimination rate constant (1/h), ^9^ standard error. ^a,b^ Rows with means of different superscripts are significantly different (*p* < 0.05).

**Table 3:** Multivariable linear regression comparing plasma half-life (hours) between MET- and MOS-treated cows.

Variable	Estimate	SE ^1^	95% CI ^2^	*p*-Value
**Treatment:**				
**MET ^3^**	Referent			
**MOS ^4^**	16.15	2.65	10.16–22.15	<0.0001
**Lactation Group:**				
**1st**	Referent			
**2nd and greater**	−3.68	2.1	−12.16	0.2
**Constant**	11.41	2.57	5.87–17.22	0.002

^1^ Standard error; ^2^ confidence interval; ^3^ subcutaneous meloxicam; ^4^ oral meloxicam.

**Table 4:** Multivariable linear regression comparing log-transformed AUC_0-∞_ (h * µg/mL) between MET- and MOS-treated cows.

Variable	Estimate ^1^	SE ^2^	95% CI ^3^	*p*-Value
**Treatment:**				
**MET ^4^**	Referent			
**MOS ^5^**	1.06	0.15	0.73–1.40	<0.0001
**Lactation Group:**				
**1 st**	Referent			
**2 nd and greater**	−0.8	0.15	−1.13–(−0.46)	<0.0001
**Constant**	3.68	0.14	3.35–4.00	<0.0001

^1^ Standard error; ^2^ confidence interval; ^3^ subcutaneous meloxicam; ^4^ oral meloxicam. Shapiro-Wilke’s test *p* = 0.65; Adjusted R2 = 0.89; Cook–Weisberg test for heteroskedasticity *p* = 0.16.

**Table 5:** Multivariable linear regression comparing maximum meloxicam plasma concentration (µg/mL) between MET- and MOS-treated cows.

Variable	Estimate	SE ^1^	95% CI ^2^	*p*-Value
**Treatment:**				
**MET ^3^**	Referent			
**MOS ^4^**	0.37	0.16	0.01–0.72	0.04
**Lactation Group:**				
**1 st**	Referent			
**2 nd and greater**	−0.56	0.16	−0.92–[−0.20]	0.007
**Constant**	1.59	0.15	1.24–1.94	<0.0001

^1^ Standard error; ^2^ confidence interval; ^3^ subcutaneous meloxicam; ^4^ oral meloxicam. Shapiro–Wilke’s test *p* = 0.18; adjusted R^2^ = 0.63; Cook–Weisberg test for heteroskedasticity *p* = 0.70.

**Table 6:** Descriptive summary of time above blood thresholds of 0.2 and 0.7 µg/mL for MET and MOS treatment groups.

	Meloxicam Plasma Concentration Threshold (µg/mL)	Mean (h)	Std. Dev. (h)	Min (h)	Max (h)
**MET**	0.7	13.5	6.8	7.0	21.0
	0.2	45.8	25.0	25.0	78.0
**MOS**	0.7	30.3	14.9	15.0	47.0
	0.2	93.0	23.7	70.0	134.0

## Supplementary Materials

The following are available online at https://www.mdpi.com/2306-7381/6/3/73/s1, Table S1: Raw plasma meloxicam levels in postpartum study cows.
